# Catching Babies, Carrying Traditions: The voices and practices of Traditional Birth Attendants in Mayuge District, East Central Uganda

**DOI:** 10.21203/rs.3.rs-6378764/v1

**Published:** 2025-04-17

**Authors:** Enid Kawala Kagoya, Proscovia Auma, Allan G. Nsubuga, Joshua Mugabi, Elizabeth Kawala, Deogratias Asabawebwa, Richard Mugahi, Brenda Mutunda, Richard Gamubaka, Agnes Namaganda, Jackline Akello, Paul Waako, Kenneth Mugabe

**Affiliations:** Busitema University; Ministry of Health; Kitibwa Restoring Hope Foundation; Busitema University; Mayuge District Local Government; Mayuge District Local Government; Ministry of Health; Busitema University; Busitema University; Makerere University; Makerere University; Busitema University; Ministry of Health

## Abstract

**Background:**

Traditional Birth Attendants (TBAs) have long played a critical role in maternal healthcare globally, particularly in rural regions of sub-Saharan Africa, including Uganda. These individuals, who often possess skills passed down through generations, provide essential maternal and neonatal care in areas with limited access to formal healthcare services. Despite their significant contributions, TBAs face challenges in managing obstetric complications and ensuring optimal maternal health outcomes due to the absence of formal medical training and limited access to resources.

In Uganda, TBAs are both recognized and banned by different health policies, creating a complex dynamic within the healthcare system. National policies have called for the regulation of TBAs and their integration into the formal healthcare system, especially in rural areas like the Busoga region, where healthcare access is limited however, the integration process has been met with various challenges, including cultural resistance, lack of training, and insufficient resources. This study aims to explore the role of TBAs in the Busoga region of Uganda, assess their practices and training needs, and evaluate the existing policies that aim to integrate them into formal healthcare settings.

**Objectives:**

To explore the role of TBAs in the Busoga region of Uganda, assess their practices and training needs, and evaluate the existing policies that aim to integrate them into formal healthcare settings.

**Methodology:**

We conducted 15 key informant interviews with traditional birth attendants in all sub-counties of Mayuge District. Key informant interviews were conducted at their respective homes/service points. This included return demonstrations from the TBAs on how they practice and perform or execute their services. Each interview lasted over an hour and was audio recorded. All recordings were transcribed and reviewed by the principal investigator. Data was analyzed using thematic analysis.

**Results:**

Some of the themes generated included how they came into existence like through Modeling from parents, apprenticeship and through assisting existing medical practitioners; what entices mothers to come to them instead of going to the facilities, specialized conditions managed and how they manage them.

**Conclusion:**

We noticed that TBA services in Mayuge district increase the risk of communities to have poor maternal and newborn health outcomes.

## Introduction

In many low- and middle-income countries, Traditional Birth Attendants (TBAs) continue to play a pivotal role in maternal healthcare, particularly in rural and underserved regions where access to formal medical facilities is limited[1]. Uganda is no exception, where TBAs are often the first point of contact for pregnant women, especially in remote areas[2]. Despite their widespread use, the training, practices, and integration of TBAs into the formal healthcare system remain critical concerns[3]. While TBAs are trusted by many communities and serve as essential caregivers, their reliance on traditional practices and lack of formal training can result in increased risks of maternal and neonatal complications[4].

Mayuge District, located in southeastern Uganda, is one such area where untrained TBAs are prevalent and continue to provide maternal care despite the potential risks associated with their informal practices[1]. This district faces significant challenges in terms of healthcare infrastructure, which makes TBAs a crucial part of the local healthcare system[3]. However, the implications of their practices, lack of training, and integration into the national healthcare framework require further examination to ensure better maternal and neonatal health outcomes[4].

Traditional Birth Attendants in Uganda generally acquire their skills informally, often through apprenticeship or experiential learning, without formal medical education or standardized training[3]. In the absence of skilled medical practitioners, particularly in rural areas, TBAs provide essential services that include antenatal care (ANC), delivery assistance, and postpartum care[1]. However, while TBAs fill the gap in maternity care, their knowledge of complications, such as eclampsia, obstructed labor, and postpartum haemorrhage, is often limited, posing risks to both the mother and the newborn[5]. Studies have shown that TBAs in regions like Mayuge District are especially vulnerable due to their lack of formal training and limited exposure to critical obstetric knowledge[6].

Despite these challenges, the role of TBAs in Uganda’s healthcare system remains a subject of debate[6].National policies have called for the integration of TBAs into formal healthcare systems to enhance maternal health outcomes, but this process has been slow and inconsistent. The Ministry of Health in Uganda has acknowledged the importance of TBAs in bridging gaps in healthcare access, especially in rural communities, but also highlighted the need for regulatory frameworks, training programs, and monitoring mechanisms to ensure their effectiveness and safety

This study aims to explore the role, practices, and challenges of TBAs in Mayuge District, focusing on their genesis, training, and integration into the formal healthcare system. Specifically, it seeks to assess the level of formal training among TBAs, identify the barriers they face, and understand the community’s reliance on them for maternal care. The ultimate goal of this study is to provide evidence-based insights that will inform national healthcare policy, specifically around the integration of TBAs into Uganda’s formal healthcare structure, to improve maternal and neonatal health outcomes

### Objectives

1. To explore the historical development and evolution of Traditional Birth Attendants (TBAs) services in Mayuge District.
2. To describe the current practices, methods, and approaches used by TBAs in managing maternal care in Mayuge District.
3. To assess the role and influence of socio-cultural factors on the pathogenesis and effectiveness of TBA services in Mayuge District.
4. To examine the challenges faced by TBAs in providing safe and effective maternal care, particularly with respect to managing obstetric complications.

### Study Site

Mayuge District is located in the southeastern region of Uganda, in the Eastern Central part of the country. It is bordered by Lake Victoria to the south, with other neighboring districts including Jinja to the northwest, Iganga to the northeast, and Kamuli to the east. The district is part of the Busoga sub-region and is divided into both rural and urban areas, with Mayuge Town Council being the district headquarters. Mayuge District has a largely agrarian economy, with most of its population engaged in subsistence farming. The population of Mayuge District is predominantly rural, with a significant proportion of the population living in villages scattered throughout the district. The rural communities often face challenges such as limited access to healthcare, education, and infrastructure. Due to its rural nature and the limited presence of formal healthcare facilities, Mayuge District relies heavily on community-based healthcare services, including Traditional Birth Attendants (TBAs) who provide maternal and neonatal care to expectant mothers. Access to skilled medical care, particularly in emergency situations, is limited, and many women in rural areas continue to rely on TBAs for assistance during childbirth.

Healthcare infrastructure in the district is underdeveloped, with a shortage of healthcare professionals, including Skilled Birth Attendants (SBAs), and inadequate access to essential maternal and neonatal health services. This has contributed to a high reliance on TBAs, whose services are seen as more accessible and culturally acceptable, despite the risks associated with their lack of formal medical training. Efforts to improve maternal health outcomes in the district are ongoing, with the government and various NGOs working to increase access to formal healthcare services, promote skilled birth attendance, and integrate TBAs into the formal healthcare system.

## Materials and methods

### Study Designs

This was a narrative qualitative study

## Materials and Methods

We conducted a qualitative study in all the sub-counties of Mayuge District. 15 in-depth interviews were conducted with at least one traditional birth attendant each sub-county. All traditional birth attendants were recruited from the community using snowball sampling. The key informant interviews were performed at the residences or premises of the traditional birth attendants following a semi-structured interview guide. Interviews were recorded with simultaneous note-taking. Deductive coding was done using Dedoose Software. All codes were categorized to generate themes and subthemes. All the parent and child codes were reviewed by the research team to make sense of them. The analysis followed a thematic analysis.

### Ethical Consideration

Ethical approval was obtained from the Mbale Regional Referral Hospital Research and Ethics Committee (MRRH-2023–342). All procedures performed in this study were in accordance with the ethical standards of the institutional research committee and with the 1964 Helsinki Declaration and its later amendments or comparable ethical standards

## Results

### Social Demographic Factors

**Table:** 

Identifier	Age	Gender	Years of experience	Location
TBA1	45 years	Female	13 years	Buguwa
TBA2	34 years	Female	5 years	Wangobu
TBA3	52 years	Female	9 years	Nawanvubu
TBA4	38 years	Female	6 years	Waitambogwe
TBA5	54 years	Female	25 years	Buwalima
TBA6	60 years	Female	35 years	kigulu
TBA7	46 years	Male	20 years	Buwaiswa
TBA8	39 years	Female	15 years	Kikuubo
TBA9	44 years	Female	11 years	Mpungwe
TBA10	32 years	Female	9 years	Bukatuube
TBA11	38 years	Female	6 years	Mayuge
TBA12	39 years	Female	12 years	Nansaga
TBA13	42 years	Female	8 years	Bukatuube
TBA14	53 years	Female	16 years	Mayuge
TBA15	62 years	Female	30 years	Mpungwe

### Thematic Areas

**Table:** 

Theme	Subtheme	Subtheme/Child Node
Historical Developments and Evolution of TBAs	Evolution	Apprenticeship
		Formal Training by MOH
		Former VHTs
		Motivation by Community
	Training Pathways	Community Training Programs
		Training by Civil society Organizations
	Training Packages	Postnatal Care
		Antenatal Care
		Delivery and childbirth
		Management of some conditions
		Helping babies breathe
Practices, methods and approaches used in the management of obstetric conditions	Identification and Diagnosis of conditions	Preeclampsia and Eclampsia.
		Birth Asphyxia
		Maternal sepsis
		Postdate (Deeni)
		Postpartum Hemorrhage
		Placenta previa
		Breech Deliveries
		Incomplete Abortions
	Conditions managed by TBA and how they are managed	Preeclampsia/Eclampsia
		Birth Asphyxia
		Postdate (Deeni)
		Nabuguma (Heat in the abdomen)
		Abortions
		Neonatal Resuscitation
		Postpartum
		APH
	Medicines used in managing particular conditions	Modern Medicines
		Traditional Medicines
	Infection Prevention and Control	**Disposal of Waste**
		Burial method
		Placenta pit
		Dispose off in the garden
		Burning
		**Personal Protective** Equipment
		Use of Gloves
		Black Kaveera
		**Handwashing practices**
		Putting soap on their hands prior to touching a patient
		Use of cooking oil
	MPDSR Process	Death Notifications
		Perinatal Death reviews
		Maternal Death Reviews
	Referral process	Referral to the health facility
		TBA to TBA referral
		Referral to Traditional Healers
Roles and influences of social-cultural factors on the pathogenesis and effectiveness of TBA services	Poverty among Mothers	Inability to afford proper medical care
	Cultural Beliefs	Ignorance about better medical care
	Husband Influence	Husband preferences
	Mother- in-low influence.	Fear of becoming widowers
	Motivation by Community	Community Influence
Challenges faced by TBAs in providing safe and effective maternal and child health care services.	Ban by the Ministry of Health	Working under fear
	Contextual factors	Failure of mothers to disclose their HIV status to them
		Lack of HIV testing Kits
		Lack of transport to take complicated cases to the Health Facility
		Harsh attitude from Health workers when they have referred complicated to the Health Facility
		Lack of space for use during management of mothers like delivery rooms and resting rooms
		Un prepared mothers for delivery
		Lack of transport to take complicated cases to the Health Facility
		Harsh attitude from Health workers when they have referred complicated to the Health Facility.
	Competition among TBAs	Lack of support from MOH
		Threats from the District Leadership
		TBA vs TBA competition
		TBA vs VHTS competition
		Competition with Private clinics
	Lack of sundries by Patients	Like gloves, Kavera etc
	Negligence of patients	Delay to reach the TBA facility
		Not taking appropriate treatment
		Poor ANC visits

### Theme 1: Historical developments and evolution of TBAs

The evolution of Traditional Birth Attendants (TBAs) had been shaped by both informal and formal learning methods. Historically, TBAs learned through apprenticeship, gaining hands-on experience from more seasoned practitioners. Over time, formal training programs led by the Ministry of Health (MOH) had helped enhance their skills. Many TBAs had prior experience as Village Health Teams (VHTs), which had provided a solid foundation in basic health care. Community support had been a significant motivating factor for TBAs, as they were often seen as vital to local healthcare, with training programs focusing on antenatal, postnatal care, and emergency childbirth practices.

#### Subtheme: Evolution of Traditional Birth Attendants

The evolution of Traditional Birth Attendants (TBAs) reflects both the changing health landscape and the enduring role of community-based birth services. Historically, TBAs were passed down through informal learning methods, primarily through apprenticeship. It was reported that TBAs often learned from older, more experienced attendants who shared their knowledge through hands-on experience rather than formal education. This apprenticeship model allowed TBAs to develop practical skills and knowledge tailored to the specific needs of the communities they served.

#### Subtheme: Apprenticeship:

TBAs reported that their initial training came through apprenticeship, where they learned directly from experienced practitioners. This informal training method allowed them to gain hands-on knowledge and skills necessary for managing deliveries, despite the lack of formal educational structures.

“Truthfully, it is as if a familial work because my paternal grandmother did the same and some paternal aunts (TBA11, Female, 38 years, Mayuge Village)”.

“My mother was a TBA she trained me, and she also delivered all of us from TBAs and me too I have never produced in the hands of a skilled health worker, (TBA 9, 44 years, Mpungwe Village)”

#### Subtheme: Formal Training by MOH:

Over time, some TBAs received formal training through the Ministry of Health (MOH) programs. This training aimed to enhance their skills and bring their practices closer to modern medical standards. TBAs highlighted that the formal training significantly improved their ability to handle complications and provided more structured knowledge.

“and now when the training came up about this, they came and looked for us and took us to Kigandalo where we were taught what we didn’t know. Like how to examine for the gestation age using the distention of the abdomen which we now try but before that we were working like we used to initially (TBA10, 32years, Bukatuube Village)”

“There were people from the ministry of Health, from Kigandalo Health Center IV in 1996. And we had two ladies, Kisiki Beatrice and Nangobi Phoebe (TBA6, 60 years, Kigulu Village)”

#### Child node: Former VHTs:

Many TBAs had backgrounds as Village Health Teams (VHTs), which gave them a foundation in basic healthcare practices, particularly maternal and child health. This prior training helped them understand the broader healthcare system, although it was not always sufficient to address more complex health conditions. Many TBAs, especially those with previous experience as former Village Health Teams (VHTs), brought valuable health experience to their roles. These former VHTs, having already been engaged in community health initiatives, found their transition into TBA work smoother. They were familiar with community dynamics and had received basic health education, which complemented their work as birth attendants.

“I am actually a VHT and while at the facility, I interact with midwives and observe how they handle these mothers, learn and also come this side and handle them using the skills gained (TBA11,38 years, Mayuge Village)”

“I was a VHT, then transitioned into a TBA with my wife after practicing at a health facility in my village, I taught my wife and she is also a TBA (TBA, 46 years,Buwaiswa Village)”

#### Child Node: Motivation by Community:

Community support was found to be a strong motivator for TBAs. They reported that their role within the community gave them a sense of purpose, and the trust placed in them by local populations fueled their continued involvement in maternal and child health care. As the need for more structured healthcare arose, the formal training by the Ministry of Health (MOH) became increasingly important. TBAs began receiving formal training that helped them learn basic medical practices and techniques in line with modern health standards. This training, often seen as essential, was intended to enhance their capabilities and reduce maternal and neonatal mortality.

“Now since that day, I stayed with the Health worker for some time, and this Hospital in Nakavule had not yet reached where it is right now, I was in Nakavule. Since then there was a health worker who invited me after she had seen that I do that work. Then going to that health worker, it was near CMS. She requested me to help keep her home where she used to go from for her work. And whenever she would go for her work, sometimes she would come back, maybe she wanted to test my ability to assist delivery, she would come back when I have delivered like three mothers and their babies are fine. So the community urged me to continue (TBA, 60 Years, Kigulu Village)”

What was particularly striking was how motivation by the community played a significant role in the evolution of TBAs. Communities supported their local TBAs not just because they were accessible, but because TBAs were seen as a vital part of the local healthcare infrastructure. People trusted these practitioners and often preferred their services due to their deep ties to the community.

“This job, I come and stay with you as house help for your children but as time goes by you tell me to stay with some of your patients. And as I observe what you are doing, when you leave the patient there with me, the patient might ask me where you’ve gone and before you know it, she has started pushing her child. By then I was still young and had not yet gotten married. Then I ask her what I should do and she tells me to go there with a thread and tie the cord 3 times. And I did so. Then she tells me to put my fingers in the baby’s mouth and I did so very fast. But for that baby she produced it while it was lying flat. So she told me to carry the baby and place it on her belly and I asked her, “won’t the baby die?” She tells no, I just put the baby on her belly. I clean her blood until she stopped bleeding (TBA14, 53 years, Bukatuube Village)”

#### Subtheme: Training pathways

TBAs engaged in community training programs that were often localized to meet the unique needs of their communities. These programs provided TBAs with essential skills that were culturally relevant and practical. Additionally, training by civil society organizations further supported TBAs by offering structured educational opportunities, resources, and guidance, which complemented their informal practices.

#### Child Node: Community Training Programs

TBAs noted that local community-based training programs were instrumental in improving their knowledge and skills. These programs often focused on maternal health topics, including antenatal care, safe delivery practices, and postnatal care, which helped bridge gaps in formal education.

“We have a community group of TBAs and we meet once in a while for a refresher and also orient the new TBAs (TBA12, Female, 39years, Nansaga Village)”

#### Child Node: Training by Civil Society Organizations

Civil society organizations played a significant role in providing training to TBAs. TBAs acknowledged that these organizations contributed to their development by offering specialized courses and resources, especially in rural areas where government support was limited

“I can’t remember which organization it was but there were some people from some organization who met at Mpuugwe primary school and took us through family planning and other things (TBA7, Male, 46 years, Buwaiswa Village)”“.

#### Subtheme: Training by their Husbands

Some TBAs acknowledged that they were trained by their husbands, who often passed on the knowledge and skills needed for childbirth assistance within the family or community. This practice of male involvement in training, though not always widely recognized, has been an essential part of the support system in certain cultures. In many cases, these husbands, often experienced midwives themselves, played a crucial role in mentoring their wives, ensuring the continuity of traditional birthing practices.

“When I came here, God tested me that my husband was one of those that used to deliver mothers from the villages this side so he taught me and I now deliver mothers and offer other services(TBA6, 60 years, Kigulu Village)” “.

“Like 20 years, as i told you my husband was initially a TBA so when I came here, when mothers used to come here, I used to also help him but he didn’t know at first that I used to do this work because by then I was still young and for him also, when they used to come here, they could just call him to come and attend to the women (TBA4, Female, 53 years, Mpungwe Village”.

#### Subtheme: Training packages

TBAs received typically focused on essential maternal and child health services. These packages included education on postnatal care, antenatal care, delivery and childbirth, management of specific conditions, and helping babies breathe the latter a critical life-saving skill for newborns facing respiratory distress. TBAs were trained to handle a wide array of situations, making them indispensable in rural and underserved areas.

“So they told us that when a mother comes when the pregnancy period is still small we check them and then tell them to return after a month. If they come when the pregnancy period is advanced, we also tell her to return because she can come just to see whether everything is fine in the womb and when you examine her you see that everything is okay then she goes but after a short while she returns with pains (TBA5, Female, 54 years, Buwalima Village”

#### Child node: Postnatal Care:

TBAs reported learning essential postnatal care practices, including monitoring maternal health after delivery and recognizing signs of complications. This training was critical in reducing maternal morbidity by providing timely intervention during the postnatal period.

“So we were taught that there are about 7 signs that we note when a mother comes. Swelling of the legs, or face, or even a mother who is not in a good condition. You send them to the facility. The legs of a sick person can be seen, mostly shiny but if you press on them the skin delays to return (TBA6, 60 years, Kigulu Village)”“.

#### Child Node: Antenatal Care:

TBAs highlighted that their training in antenatal care focused on identifying early signs of pregnancy-related complications, counselling mothers on proper nutrition, and encouraging health-seeking behaviors. This proactive care helped improve pregnancy outcomes, especially in rural areas with limited access to formal health services.

“Really what they told us is what I do. She comes with gloves and a polyethene sheet. The polyethene sheet is place on the bed where she is going to lay while I use the gloves to examine her. Even when I am examining the abdomen alone I put on gloves. So if I need to examine the other parts she carries extra pairs of gloves (TBA4, Female, 38 years, Baitambogwe Village)”.

“We used to have the room and even the polyethene sheets so that when someone comes when they are badly off, we just tell them to have a sit and we work on them but now that no longer works(TBA7, Male, 46 years, Buwaiswa Village)”

#### Child Node: Delivery and Childbirth:

Training in delivery and childbirth equipped TBAs with the skills to assist in safe deliveries. They shared that the training provided them with essential techniques for managing deliveries, though their ability to handle complicated cases remained limited without access to medical facilities.

“Now the mother comes with her pregnancy and requests for a checkup and I check her while putting on my gloves. Because God helped me that I saw what I was taught and still see it as if I first saw them yesterday. Now you can see the baby in the womb and how it is lying and if possible, I deliver here from here (TBA8, Female, 39 years, Kikuubo Village)”

#### Child Node: Management of Some Conditions:

TBAs also received training in managing specific maternal and neonatal conditions, including complications like preeclampsia, hemorrhage, and neonatal resuscitation. They emphasized that while the training improved their capacity to manage these issues, referral to health facilities was often necessary for severe cases.

“And if someone has problems in their pregnancy we direct them in the traditional way and God willing the problem is curbed. People come and say that they have wounds in their private parts when they are pregnant we know that is syphilis (TBA3, Female, 52 years, Nawanvubu Village)”.

#### Child Node: Helping Babies Breathe:

TBAs mentioned that specialized training in neonatal resuscitation, specifically in helping babies breathe after birth, was crucial in reducing neonatal mortality. This training gave them the confidence to intervene in cases of asphyxia, although resources for such interventions were often limited.

“They taught us how to deal with babies, especially newborn babies born when they are tired and can’t breathe (TBA9, Female, 44years, Mpungwe Village)”

#### Theme: Practices, methods, and approaches used in the management of obstetric conditions

TBAs had employed a range of methods to identify and manage obstetric conditions such as preeclampsia, birth asphyxia, and postpartum hemorrhage. They diagnosed and addressed complications through a mix of traditional knowledge and modern medical practices. Conditions like preeclampsia, birth asphyxia, and postdate pregnancies had been managed through observation, interventions, and referrals when necessary. TBAs also used both modern medicines and traditional remedies to treat conditions, making them essential in areas with limited access to formal healthcare.

#### Subtheme: Identification and Diagnosis of Conditions

TBAs identi**fi**ed and diagnosed conditions such as preeclampsia, birth asphyxia, and postpartum haemorrhage based on physical symptoms. They reported using traditional knowledge, such as observing swelling or hypertension, but noted that advanced diagnostic tools were often unavailable.

“People come and say that they have wounds in their private parts when they are pregnant we know that is syphilis. They tell you that “endira” is paining (TBA 5, Female, 54 years, Buwalima Village)”

How do you usually manage Syphilis?

“I use herbs. I might know them but not by name. I might know that this herb and that one when mixed and cook them they can treat this condition (TBA 5, Female, 54 years, Buwalima Village)”

But how do they look like

“They are of wood or husks(TBA 5, Female, 54 years, Buwalima Village)”

Husks like for sweet potatoes?

“No husks of trees mostly those we have around this village (TBA 5, Female, 54 years, Buwalima Village)”

Now how do you distinguish between the ones you require for a certain treatment?

“I base on what I was told by the people who introduced me to the practice. If I was told that this one is used for this then I know it for that use. So when it is the first time for the mother to come here I take my time to examine her well and know the baby’s status. This is what I use (shows a fetospcope)to know according to the fetal lie and orientation, even the step the baby is in I know it is for pass out. Even the way she is having her pains you know it time (TBA 5, Female, 54 years, Buwalima Village)”

#### Child node: Preeclampsia

Preeclampsia was one of the conditions that TBAs were particularly focused on diagnosing, though the study indicated that TBAs often lacked the tools required for a definitive diagnosis. TBAs in the study reported identifying preeclampsia through observable symptoms such as high blood pressure, swelling in the hands and feet, and protein in the urine. They often relied on community-based knowledge and experience to recognize these signs. However, TBAs noted that they were limited in their ability to fully manage preeclampsia due to a lack of medical equipment like blood pressure cuffs and urinalysis kits. While some TBAs attempted to offer basic interventions, including encouraging rest and hydration, they often referred cases with more severe symptoms to health facilities for further management. The study found that in the absence of formal healthcare, TBAs were often the first line of defense, but they expressed concern that without proper training, they could not always identify the condition early enough to prevent complications such as eclampsia or stroke.

“We treat those here using traditional medicine. We use paraffin mixed with tea leaves and dust and we scrub her and then cover her and then cover her with a mat (TBA10, Female,32 years, Bukatuube Village)”

Do you cover her once or cover in intervals?

“I cover her and when I see she is still shivering I cover her again. Now for me I usually have my Panadol in the house so if she is still shivering I give her some tea and some 2 Panadol tablets which in most cases helps to relieve the shivering. I don’t know how it works but it works for me (TBA10, Female,32 years, Bukatuube Village)”

Yes, because as I have told you, when someone comes with her condition, I just get the herbs and give to them to drink and bath and the become better (TBA10, Female,32 years, Bukatuube Village)”

#### Child Node: Birth Asphyxia

Birth asphyxia, another common condition encountered by TBAs, was identified primarily through signs such as a lack of breathing or a weak cry from newborns immediately after birth. TBAs in the study shared that they used their training in neonatal resuscitation, where they would attempt to stimulate the baby through methods such as rubbing the chest or using the “mouth-to-mouth” method. However, the study found that TBAs’ interventions for birth asphyxia were sometimes limited by a lack of access to proper resuscitation equipment, such as neonatal resuscitators or oxygen. As a result, the ability of TBAs to manage severe cases of birth asphyxia effectively was compromised. Despite these challenges, TBAs emphasized their role in ensuring that newborns with asphyxia received immediate attention and, when necessary, referred them to health facilities for more advanced care. The study concluded that while TBAs were skilled in recognizing and providing initial interventions for birth asphyxia, their ability to manage it effectively was constrained by inadequate resources and lack of training in advanced neonatal care.

“When mothers come when they are tired, I first give them first aid of a cup of cold hot then I relax them on the bed and wait for the outcome (TBA15, Female,62 years, Mayuge Village)”

So what happens after?

“If all goes well, they give birth well and we thank God but most times if they fail, I call a boda boda and they take them to Iganga,(TBA15, Female,62 years, Mayuge Village)”

#### Child Node: Maternal Sepsis

Maternal sepsis was another condition that TBAs in the study identified and attempted to manage, particularly in cases where women experienced infections after childbirth. The study found that TBAs were able to recognize early symptoms of sepsis, such as fever, foul-smelling discharge, and abdominal pain. They often used traditional remedies, such as herbal concoctions, to try and alleviate symptoms, though these were not always effective in preventing or treating infections. TBAs also noted that they would encourage women to seek further care if the symptoms worsened or if the woman appeared very ill. However, the study revealed that many TBAs lacked a full understanding of the severity of sepsis and the importance of timely antibiotic treatment. In cases where the condition was severe, some TBAs referred women to health facilities for treatment, though this was often complicated by delays in accessing transportation or reluctance from patients to visit health facilities. The study highlighted that the lack of medical knowledge and access to antibiotics made it difficult for TBAs to effectively manage maternal sepsis, which could lead to increased morbidity and mortality if not promptly addressed.

“We use certain herbs and plants that have antimicrobial properties. For example, we use plant-based concoctions or teas made from roots, leaves, or bark to fight infections and reduce inflammation (TBA9, Female, 44 years, Mpungwe Village)”

“Warm compressions or poultices made from locally sourced herbs or other substances are applied to the abdomen or other affected areas to soothe pain and potentially promote healing(TBA9Female, 44, Years, Mpungwe Village)”“.

“Spiritual or ritual healing practices are employed, such as prayer, cleansing rituals, or seeking the help of a traditional healer or midwife to restore balance and health (TBA6. Female,60 years, Kigulu Village)”“.

“We also use steam or herbal baths as a method to help the body “sweat out” the infection, as they help with detoxification and fever reduction(TBA11, Female, 38 years, Mayuge Village) ““.

#### Child Node: Postdate (Deeni)

In the study, TBAs often encountered pregnancies that extended beyond the expected delivery date, a condition commonly referred to as *Deeni*. This was frequently diagnosed by TBAs based on their experience with expected delivery timelines and their observation of the mother’s condition. TBAs would monitor for signs of labor and advice women to prepare for delivery once the pregnancy exceeded the normal gestational period. While some TBAs used traditional methods to try to hasten labor, such as herbal teas or abdominal massage, they also recognized the risks associated with postdate pregnancies, such as an increased risk of stillbirth or birth defects. When TBAs identified a postdate pregnancy, the study found that they frequently referred the woman to a health facility for further monitoring and possible induction of labor, especially if there were additional risk factors involved. TBAs stressed that they were not always able to intervene in a meaningful way for postdate pregnancies, as the condition required more advanced medical attention. However, their knowledge of *Deeni* allowed them to recognize the condition early enough to suggest that women seek proper medical care.

“We use some herbs like **Enfwoddo** which tare the uterus and are used to stimulate contractions or prepare the body for labor (TBA14, Female, 53 years, Mpungwe Village)”“.

**“We** offer techniques such as abdominal massage, pressure points, or gentle stretches to help the baby move into a favorable position and stimulate the onset of labor (TBA1, Female, 34 years, Buguwa Village)”“.

“Emotional and spiritual guidance is offered to reduce stress and encourage relaxation, with the belief that a calm, balanced state can help trigger labor (TBA9Female, 44 years, Mpungwe Village)”“.

#### Child node: Postpartum Hemorrhage

Postpartum hemorrhage (PPH) was another condition that TBAs encountered frequently, especially in the aftermath of childbirth. The study found that TBAs identified PPH primarily through visible signs such as excessive bleeding from the birth canal after delivery. Many TBAs had learned from experience that excessive bleeding could be life-threatening and required immediate attention. In response, some TBAs attempted to manage PPH by encouraging the mother to breastfeed, which could help with uterine contraction and reduce bleeding. Some also used traditional herbal remedies to control bleeding. However, the study found that many TBAs lacked access to more effective medical interventions, such as uterotonics or blood transfusions, which are essential in the management of severe hemorrhage. In cases of severe PPH, TBAs often referred women to health facilities for more advanced care. The study highlighted that while TBAs were able to recognize and manage mild cases of PPH, they struggled to handle more severe cases effectively due to limited resources and training.

“I have been lucky all my years of practice. Allah has so far helped me I have never had such but if she is done giving birth I usually tell her that if there’s any problem she should tell me because I will not check you like I am checking a young person. So she should tell me so that we see what could be wrong (TBA9, Female, 44, years, Mpungwe Village)”“

“If that happens I tell the husband or the person she has come with to get a boda-boda and they take her to Buwaiswa (TBA9, Female,44, years, Mpungwe Village)”“

#### Subtheme: Conditions managed by TBA and how they are managed

TBAs managed a range of conditions, including preeclampsia, birth asphyxia, postdate pregnancies, and neonatal resuscitation. They used a combination of observation, traditional remedies, and modern medical interventions, although more complicated cases required referrals to health facilities.

#### Child node: Preeclampsia/Eclampsia

The study found that Traditional Birth Attendants (TBAs) managed conditions like preeclampsia and eclampsia through a combination of observation, symptom recognition, and, in some cases, traditional remedies. While TBAs were aware of the signs of preeclampsia such as high blood pressure and swelling they were not always equipped to handle the severe complications that could arise from these conditions. The study revealed that TBAs often referred women with suspected preeclampsia or eclampsia to health facilities, as these conditions required medical intervention that TBAs were not trained to provide. However, some TBAs employed traditional methods such as herbal remedies or dietary adjustments to alleviate symptoms, even though these approaches were not always effective. This demonstrated a gap in their training and resources, as they were sometimes forced to manage such critical conditions without the appropriate medical support.

“Now while managing amakilo, I use those potato leaflets. When I tell you this you might despise the leaflets but they are medicinal(TBA9Female, 44, Years, Mpungwe Village)”.

“I know the leaflets and have heard of their potential. You pour the leaflets in her head if she is shivering, because there are 2 types of “amakilo”, there is where the girl doesn’t bathe the herbs. For that you give her the leaflets only without even anything else (TBA9Female, 44, Years, Mpungwe Village)”.

So how does she use them?

“She squeezes them and uses it to wash her head(TBA9Female, 44, Years, Mpungwe Village)”“.

For how long and how many times?

“Even if it’s just that one time(TBA9Female, 44, Years, Mpungwe Village)”“

#### Child Node: Birth Asphyxia

The study revealed that TBAs often encounter birth asphyxia, a condition in which the baby does not receive enough oxygen during labor and delivery. TBAs typically managed birth asphyxia by performing neonatal resuscitation using traditional methods such as clearing the airways with suction, stimulating the infant, and ensuring proper positioning of the baby to aid breathing. However, the study found that the success of these interventions was limited by the TBAs’ lack of access to more advanced resuscitation tools like oxygen or neonatal ventilators. In cases of severe asphyxia, TBAs frequently referred the baby to health facilities for further management, though transportation challenges and delays in seeking care were identified as obstacles that reduced the chances of successful outcomes. TBAs, while aware of the urgency of birth asphyxia, were constrained by their training and the lack of medical resources to provide effective neonatal care.

“Now for me I think these two people getting tired is due to you as the birth attendant if she is not just sick. As you tell her to push on every contraction, it makes both the mother and the baby tired. It requires you to instruct her to push when you are sure the baby is ready and within a few pushes 4, 5 pushes the baby I out but if you put her through those things of pushing over and over, you even leave her there and first do other things, you might come back when she is tired and she might even rapture(TBA9, Female, 44 years, Mpungwe Village)”“.

Now how do you gauge that this is a weak contraction or this contraction is sufficient and the other is insufficient to prevent getting tired?

“So for me if she comes in labor, I ask for gloves, I examine her and tell her that her baby is still far so I tell her to wait to push(TBA2, Female, 52 years, Nawanvubu Village)”

What I want to know is how you count those steps

‘You might check and note that the head is still up or you might tell her to push because you think the baby is near but in actual sense she is still far. So, you check and then you tell to first rest. After some time, you notice the head has reached and then tell her to push and after a few pushes the baby is out(TBA13, Female, 42 years, Bukatuube Village)”t’

“Also for those you give her the leaflets and a little paraffin. She uses it to wash her head and she gets relieved(TBA14, Female, 53 years, Mpungwe Village)”“

So you use the sweet potato leaflets

Yes

You drop in some little paraffin and that problem is relieved and if it persists and it seems that you won’t manage, you send her to the hospital(TBA6,Female, 60 years, Kigulu Village)”

#### Child Node: Postdate (Deeni)

The management of postdate pregnancies (referred to as *Deeni* in the study) was a common issue for TBAs, as many women in rural areas had limited access to prenatal care or ultrasound to assess the gestational age of the pregnancy. TBAs typically diagnosed postdate pregnancies based on maternal reports or physical signs such as the size of the belly or absence of labor at the expected due date. For these cases, TBAs often encouraged women to be patient, offering traditional advice on easing labor, such as using herbal teas or specific dietary recommendations. In some instances, TBAs attempted manual interventions to encourage labor, though these practices were not always effective and sometimes posed risks to both the mother and baby. The study found that TBAs could not perform medical procedures such as induction of labor and often referred women with prolonged pregnancies to health facilities for further evaluation and management.

“Now for you who is not God, you don’t engage in such. Those are Godly issues. Because I may do it and you find a patient somewhere and you tell them you found a Musawo somewhere who can work her and when reach here, I tell you I can’t work on her. Is that even acceptable?. So that I can’t do, I don’t know them (TBA1,, Female, 45 years, Buguwa Village)”

#### Child Node: Nabuguma (Heat in the Abdomen)

Nabuguma, or “heat in the abdomen,” was reported by TBAs as a common condition that they managed during pregnancy and childbirth. This condition was often perceived as a result of excessive internal heat, which could be caused by various factors, such as stress or physical strain. The study found that TBAs would treat “nabuguma” using traditional methods such as herbal remedies, warm compresses, or abdominal massages. While these practices were widely believed to alleviate discomfort, the study noted that there was limited evidence to support the effectiveness of these methods in treating underlying causes. TBAs commonly relied on their knowledge passed down from previous generations, but without proper diagnostic tools, it was difficult to ascertain whether the symptoms were caused by more serious medical conditions, such as infection or complications related to pregnancy. As such, TBAs sometimes referred women experiencing nabuguma to health facilities if they suspected the symptoms indicated a more serious health issue.

“If you’re loosing a pregnancy at around 3 months or 4, or you’ve lost like 5, I give you those herbs and you take them and we see what we can do, the pregnancy grows until you can give birth (TBA15, Female, 62 years, Mayuge Village)”

Now for those herbs, for how long does one take them? How many times does one take them a day?

“Twice only a day(TBA15, Female, 62 years, Mayuge Village)”

For how long?

“For about a month only (TBA15, Female, 62 years, Mayuge Village)”

After that month one is better? The pregnancy grows well?

“Yes ma’am (TBA15, Female, 62 years, Mayuge Village)”

#### Child Node: Abortions

The study found that TBAs were sometimes called upon to manage cases of spontaneous or induced abortions. In rural areas, where access to family planning services was limited, TBAs often played a central role in assisting women who experienced miscarriage or attempted abortions. For spontaneous abortions, TBAs typically provided emotional support and assisted with the physical recovery, sometimes using herbal remedies to help with bleeding or pain management. However, in cases of induced abortions, TBAs were occasionally involved in the process, especially when women sought their help to terminate pregnancies. This raised significant concerns regarding the safety and legality of abortion practices. While TBAs attempted to offer support, the study highlighted that they lacked the medical training to manage complications from abortions effectively, and many women were at risk of infections or other health issues due to unsterile practices and improper care.

“If you’re losing a pregnancy at around 3 months or 4, or you’ve lost like 5, I give you those herbs and you take them and we see what we can do, the pregnancy grows until you can give birth (TBA15, Female, 62 years, Mayuge Village)”.

“When I notice she is in pain. And if she is in pain, and she thinks she is going to come here where I am like how I assist them in these miscarriages(TBA9, Female, 44 years, Mpungwe Village)”

#### Child Node: Neonatal Resuscitation

Neonatal resuscitation was a critical aspect of TBA practice, especially for those who attended home births. The study found that many TBAs were trained in basic neonatal resuscitation techniques, such as clearing the infant’s airways, stimulating the baby to breathe, and positioning the baby correctly after delivery. However, these methods were often rudimentary, and TBAs lacked the equipment and expertise to perform more advanced resuscitation techniques, such as the use of oxygen or mechanical ventilation. TBAs reported that their success with neonatal resuscitation depended heavily on the timing of the intervention and the severity of the asphyxia. Although they managed to save many babies with basic techniques, the study emphasized that the lack of formal training and medical resources put both the neonates and mothers at risk, particularly in cases of severe birth asphyxia or respiratory distress.

“If God allows and the baby comes out well, I get her out and hurry up to cut the chord then hold her by the legs and buttocks putting her upside down, and take some time while still holding her and you notice she starts breathing. When she breathes for the second time you know you are good. Usually I even first leave the mother and tell her to cover herself so that she doesn’t become cold as I focus on the baby. The baby can have like 2–3 breaths and she starts crying, then you know that life is back. Then you cover her very well And you put her aside and then go back to the mother and remove the remaining things(TBA15, Female, 62 years, Mayuge Village)”

So putting the baby upside down helps in breathing

“Yes”

What of squeezing the buttocks?

“It helps the baby not to let air pass through but to remain in because might breath once and it passes and she dies (TBA15, Female, 62 years, Mayuge Village)”

Now these mothers after giving birth the children are assessed according to their breathing like at 1 minute, 5 minutes do you do that?

“No for us we are usually doing this and that but also because for us we didn’t learn them very well. But also, you might be holding the baby and she slips and falls that is why we only try putting her upside down (TBA15, Female, 62 years, Mayuge Village)”

But she gives birth to an exhausted baby from here and you don’t want it to die, what do you do?

“You it upside down and beat the legs (TBA15, Female, 62 years, Mayuge Village)”

Like how this one was turn the legs the other side?

No you put the legs up and you hit gently on the legs like this and it breathes (TBA15, Female, 62 years, Mayuge Village)”

For how long

“Utmost 20minutes(TBA15, Female, 62 years, Mayuge Village)”

Have you ever done it and it refused to work?

“No it has never (TBA15, Female, 62 years, Mayuge Village)”

#### Child node: Prenatal care

The study also reported that traditional birth attendants (TBAs) played a significant role in providing prenatal care to expectant mothers in Mayuge district. These TBAs, often part of the local community, are trusted by many due to their cultural familiarity and experience in childbirth. In Mayuge, where access to formal healthcare facilities may be limited, these attendants serve as the primary source of care for pregnant women, offering services such as health assessments, advice on diet, hygiene, and the monitoring of the pregnancy’s progress. While the quality and extent of care provided by TBAs may vary, their presence highlights a key reliance on traditional practices alongside the formal healthcare system. The study suggests that, despite the growing push for professional medical care, the role of TBAs remains crucial in rural areas like Mayuge, where they bridge gaps in the healthcare system

“As long as she is still here with me, she can take tea like 2 to 3 times because sometimes she might be in labor but at the sometimes yawning and clearly see that she is hungry. so I give her a cup of tea with something to accompany the tea and when you see her situation is not becoming better you send them to the health center. And for me nowadays I no longer keep people here for long maybe if its late at night without transportation (TBA15, Female, 62 years, Mayuge Village)”

“I use my hands to feel and know the baby’s lie and you can know the normal and abnormal lie. Even when she comes and doesn’t know the period of pregnancy, you can know and tell her that she will give birth in a certain month (TBA9Female, 44, Years, Mpungwe Village)”“.

#### Child Node: Postpartum Care

The study found that TBAs were primarily responsible for managing postpartum care, which included monitoring the mother for signs of complications such as postpartum hemorrhage (PPH), infection, and uterine involution. TBAs provided counseling and physical care, such as massaging the abdomen to help the uterus contract and expel remaining placenta, as well as helping the mother with breastfeeding. In the event of complications like PPH, TBAs used traditional remedies, such as herbal concoctions to control bleeding, although these methods were not always effective. The study found that when signs of severe postpartum complications were detected, such as excessive bleeding or fever, TBAs referred women to health facilities for further treatment. However, the study also noted that delayed referrals due to transportation challenges or reluctance from the women’s families were often significant barriers to ensuring timely and effective postpartum care.

“Rituals, prayers, or ceremonies are performed to bless the new mother, protect her from negative energy, and encourage a smooth transition into motherhood. Family members and community support play a key role in the healing process (TBA12, Female, 39 years, Nansaga Village)”.

*“After childbirth, herbal baths made from leaves, roots, or flowers are commonly used to cleanse and heal the body. Herbs like* **rosema*r*y, g*i*nge*r*** *etc aid in wound healing, improve circulation, and help with relaxation(TBA9Female, 44, Years, Mpungwe Village)”*.

#### Child Node: Delayed Cord clamping

Many birth attendants reported that they allow the umbilical cord to remain intact until it naturally stops pulsating. They believe that waiting for the pulse to cease allows the baby to receive the full benefits of the remaining blood in the placenta. Others said that they wait for the cord to “detach” naturally, either through gravity or when the baby has moved away from the mother, before cutting it. This symbolized the bond, between mother and child and give the baby time to adjust to the outside world.

“When cutting the chord show long do you take before cutting it?”

“To be honest I can’t tell you how long I take because as I am attending to the mother I usually don’t look at the time. But as the child gets out and I put her on the mother’s belly it’s an indicator for me to tie and cut the chord”

#### Child Node: APH (Antepartum Hemorrhage)

Antepartum hemorrhage (APH), which involves bleeding before labor, was another condition that TBAs reported managing. The study found that TBAs were aware of the potential dangers of APH, but they had limited capacity to diagnose and manage the condition effectively. TBAs typically relied on observation of symptoms such as vaginal bleeding and abdominal pain to identify APH. However, they often lacked the medical knowledge to differentiate between the types of bleeding (placenta previa, placental abruption) and their potential risks to the mother and baby. TBAs reported that they generally advised women with APH to seek care at a health facility, but the study revealed that transportation delays or a lack of trust in formal healthcare systems sometimes delayed or prevented timely referrals. As a result, some women experienced poor outcomes, including stillbirths or maternal complications, because APH was not managed appropriately.

#### Subtheme: Admissions

Traditional Birth Attendants acknowledged that they offer admissions to mothers who present with some conditions like delayed contractions, birth Asphyxia etc

“Talking about the times you used to operate from home when they come like you’ve said maybe at night; do you provide accommodation for all of them or the man goes back and you remain with the patient only(TBA9Female, 44, Years, Mpungwe Village)”?

“The man usually goes back home and I remain with one caretaker and the patient

So do have where they sleep?

“They just sleep in the sitting room there (TBA9Female, 44, Years, Mpungwe Village)”

No if she has slept there is there any help you give them maybe medicine for this time or anything?

“I give tea and she take because you don’t know where she has come from whether she has eaten (TBA9Female, 44, Years, Mpungwe Village)”

#### Subtheme: Cord around the neck

“Now for me in my understanding because those babies surely disturb but when the head is out try unwrapping the chord and then tell the mother to push. Because when it’s in the neck whenever she pushes and rests the baby goes back even when the head is out so whenever she pushes I unwind and after a few times it releases. Because if you wait for the baby to come out it might injure it and so me I have a way I unwind it. When the baby is out I put her on the mother’s belly then I start tying the chord(TBA9Female, 44, Years, Mpungwe Village)”.

“Now how do you know that this mother has a baby with the chord in the neck as you monitor them before they are term?(TBA9Female, 44, Years, Mpungwe Village)”

“When the baby is still inside I can’t tell. I just see when the baby is out.(TBA9Female, 44, Years, Mpungwe Village)”

“You pull it out and it comes. You don’t pull it itself but you pull the baby if the baby has come, you can easily remove, tie and cut the chord and the baby becomes better(TBA9Female, 44, Years, Mpungwe Village)”

#### Child Node: Medicines Used in Managing Particular Conditions:

TBAs used both modern medicines and traditional remedies to treat obstetric conditions. They reported using herbs, as well as pharmaceutical drugs like antibiotics, to manage infections or complications. However, TBAs noted that a lack of access to modern medical supplies sometimes hindered their ability to provide effective treatment.

“So they told us that when a mother comes when the pregnancy period is still small we check them and then tell them to return after a month. If they come when the pregnancy period is advanced, we also tell her to return because she can come just to see whether everything is fine in the womb and when you examine her you see that everything is okay then she goes but after a short while she returns with pains(TBA8, Female,38 years, Kikuubo Village)”.

“We used to have the room and even the polyethene sheets so that when someone comes when they are badly off, we just tell them to have a sit and we work on them but now that no longer works. everyone must carry their own polyethene because if now someone comes when she is badly off without her own polyethene and we work on her with our own and she goes to Buwaiswa (TBA12, Female, 39 years, Nansaga Village)”

#### Subtheme: Management of obstetric conditions

TBAs applied a variety of traditional and learned methods. One of the first steps was the identification and diagnosis of conditions. TBAs became adept at recognizing signs of preeclampsia, birth asphyxia, maternal sepsis, postdate pregnancies (Deeni), and postpartum hemorrhage. Identifying these conditions early was critical for ensuring appropriate responses. For example, it was noted that TBAs often relied on physical symptoms and community knowledge to diagnose these potentially dangerous conditions.

“Now the mother comes with her pregnancy and requests for a checkup and I check her while putting on my gloves. Because God helped me that I saw what I was taught and still see it as if I first saw them yesterday. Now you can see the baby in the womb and how it is lying (TBA9Female, 44, Years, Mpungwe Village)”.

#### Subtheme: Specific conditions managed

TBAs had a broad scope of practice. Preeclampsia and eclampsia were managed through careful observation and, in some cases, immediate referrals to higher-level care facilities. Birth asphyxia often required quick intervention, including neonatal resuscitation techniques that TBAs were trained to administer. For conditions like postdate pregnancies (Deeni), TBAs used observation and advised referrals for further medical care.I examine like how they examine the abdomen

Now for me I don’t know how they examine the abdomen so can you demonstrate for us?(TBA9Female, 44, Years, Mpungwe Village)”

Now the mother comes with her pregnancy and requests for a checkup and I check her while putting on my gloves. Because God helped me that I saw what I was taught and still see it as if I first saw them yesterday. Now you can see the baby in the womb and how it is lying(TBA9Female, 44, Years, Mpungwe Village)”.

What do you use to see? Do you use your naked eyes?

“I use my hands to feel and know the baby’s lie and you can know the normal and abnormal lie. “Even when she comes and doesn’t know the period of pregnancy, you can know and tell her that “she will give birth in a certain month(TBA9Female, 44, Years, Mpungwe Village)”.

So you only use your hands?

“yes only in cases of listening to the baby’s heart do I use a device(TBA9Female, 44, Years, Mpungwe Village)”

So what do you use to listen to the fetal heart?

“We were given some devices but mine got broken so now I don’t have(TBA9Female, 44, Years, Mpungwe Village)”.

So or instance today if I come and I need to know my fetal heart what do you use?

I just do the checkup minus the fetal heart rate and that doesn’t bother me because the health Centre is nearby. And still as you examine you can differentiate a live and dead baby because as you examine you feel the baby also moving in the womb(TBA9Female, 44, Years, Mpungwe Village)”.

Other conditions that TBAs managed included Nabuguma, a traditional term for “heat in the abdomen,” and abortions, both of which were addressed using local methods and sometimes herbal remedies. Postpartum care was another area where TBAs played a key role, offering support to mothers after childbirth to ensure recovery and address any complications. Additionally, they managed Antepartum Hemorrhage (APH), which required quick responses to prevent excessive bleeding.

#### Subtheme: Medicines used in the management conditions

TBAs employed a mix of modern medicines and traditional remedies. Modern medicines like antibiotics were used when needed, especially for conditions like maternal sepsis, while traditional medicines, such as herbal remedies for various conditions, were common. This blend of practices helped TBAs navigate a wide range of maternal health challenges.

“Yes, I just try but it’s not the ideal. I try it to stop the coldness and could be causing the shivering (TBA9Female, 44, Years, Mpungwe Village)”

“Going back to “amakilo” what do you think brings them and how does paraffin help to relieve the shivering?”

“I don’t know. As I said, I just crammed it that way and after trying it, it seemed to work, reliving the symptoms.(TBA9Female, 44, Years, Mpungwe Village)”

“We also use oxytocin especially when mothers take long while to get contractions and we five like one bottle on the thigh(TBA9Female, 44, Years, Mpungwe Village)”.

#### Subtheme: Infection Prevention and Control

Infection prevention and control were critical components of safe delivery practices. TBAs practiced various methods of waste disposal, which included traditional techniques like burial, using a placenta pit, or even disposing of waste in the garden. These practices, although not always ideal, were rooted in local customs and the resources available to TBAs. In some cases, waste was burned as a way to reduce contamination risks.

#### Subtheme: Disposal of Waste:

TBAs employed various waste disposal methods, including burial, burning, and using designated pits for placenta and other biohazardous materials. They indicated that these methods were used to reduce the risk of infections, although the effectiveness of these methods varied.

“I used to have a pit where I used to put them. When someone comes for me or comes here directly since sometimes one can just come with the mother here maybe because they don’t have another person to take care of her as he comes for me. So I go and dig my pit which I know won’t be dug up by hens where I put the “kitani (TBA7 Male, 46 years,Buwaiswa Village)”.

#### Subtheme: Personal Protective Equipment:

The use of gloves, black plastic bags, and other personal protective equipment (PPE) was common among TBAs to reduce the risk of contamination. They reported that although PPE was useful, access to these materials was often inconsistent, which affected their ability to maintain proper hygiene.

You come with gloves, Bed sheets, polythene sheet razor blade and the sheets for the baby (TBA9, Female, 44 years, Mpungwe Village)”

#### Subtheme: Handwashing Practices:

TBAs emphasized the importance of handwashing before attending to patients. They reported using soap and sometimes cooking oil to clean their hands before handling expectant mothers, which they believed helped prevent the spread of infections.

“As you know, this is a village, a patient can come when you are just from the garden, you just put on the gloves and rescue her (TBA7 Male, 46 years,Buwaiswa Village)”.

“I wash my hands if there is water nearby but the priority is to save a life first (TBA14, Female, 53 years, Mpungwe Village)”.

In terms of personal protective equipment, TBAs commonly used gloves and black kaveera (plastic bags) to handle contaminated materials, which helped maintain hygiene. Handwashing practices were another essential component; TBAs were observed to use soap before any patient interaction, ensuring basic hygiene. Interestingly, some TBAs also used cooking oil as an additional protective measure for their hands.

“I use the gloves to examine her. Even when I am examining the abdomen alone I put on gloves. So if I need to examine the other parts she carries extra pairs of gloves (TBA1, Female, 45 years, Buguwa Village)”.

“Everyone must carry their own polyethene because if now someone comes when she is badly off without her own polyethene and we work on her with our own and she goes (TBA3, Female, 53 years, Nawanvubu Village)”

#### Subtheme: MPDSR Process

The study found that TBAs were involved in the Maternal and Perinatal Death Surveillance and Response (MPDSR) process, which is an important component of monitoring and improving maternal and neonatal health. TBAs played a role in reporting deaths and complications during childbirth, though their participation was often informal. They provided death notifications when a maternal or perinatal death occurred, which were typically submitted to the local health authorities. However, the study noted that TBAs lacked the formal training to conduct in-depth reviews of these deaths, and often relied on community-based leaders or health workers to gather more detailed information about the circumstances surrounding these deaths. Despite these limitations, TBAs expressed a desire to contribute to improving maternal and neonatal care by providing feedback and sharing information about the deaths they encountered. TBAs often communicated their observations to healthcare providers, who could then investigate the causes and contribute to the development of strategies to prevent future deaths.

“I have never lost a mother but I have lost like two babies because the other one, she gave birth well but after some time the baby started coughing and towards the morning it became worse so I sat on the bicycle with the baby and took him to the Buwaiswa, on reaching he was dead. And the other one came here in her labor, on giving birth the baby’s head had no bone so I think the baby was born dead. Otherwise, i have not lost any other babies (TBA3, Female, 52 years, Nawanvubu Village)”.

#### Subtheme: Death Notifications

TBAs were responsible for notifying the community and sometimes local authorities about maternal or perinatal deaths, but the process was not always systematic. In many instances, TBAs would inform family members first, who would then report the death to health authorities if necessary. The study highlighted that TBAs were not always included in the formal death notification processes, as their involvement was sometimes seen as informal or not officially recognized. This created a gap in the flow of information, leading to delays in reporting deaths to the appropriate health authorities. As a result, the data collected on maternal and neonatal deaths might not have been as complete or timely as required for effective surveillance and response.

“I have never lost a mother but I have lost like two babies but I only reported to the relatives and told them to pic their dead bodies (TBA15, Female, 53 years, Mayuge Village)”

#### Subtheme: Perinatal Death Reviews

The study also noted that TBAs were somewhat involved in perinatal death reviews, though they were generally not the primary agents for conducting such reviews. TBAs reported that they were occasionally called upon by health workers or local community leaders to provide input on the factors surrounding perinatal deaths that occurred in their care. However, the study revealed that TBAs often lacked the training to conduct thorough reviews, such as gathering detailed clinical data or identifying systemic issues in maternal care that might have contributed to the death. TBAs shared their experiences with families and other community members, but it was often up to health professionals to conduct formal perinatal death reviews and determine causes and prevention strategies.

“We don’t review anything, those days when we were working peacefully, we would report to the nearby health facility and they review but they even don’t tell us the cause of death (TBA12, Female, 39 years, Nansaga Village)”.

#### Subtheme: Maternal Death Reviews

Similarly, TBAs played a limited role in maternal death reviews, though they were sometimes included in community discussions about the causes of these deaths. In rural areas, TBAs often knew the circumstances surrounding the deaths they attended, but the study found that TBAs lacked access to formal data or resources to systematically review maternal deaths. When asked about maternal death reviews, TBAs expressed a desire for more training and inclusion in the process. They believed that their hands-on experience could offer valuable insights into the causes of maternal deaths in their communities. The study highlighted the need for TBAs to be more actively involved in maternal death reviews, as they are often the first point of contact for women in labor and are likely to have useful information about the circumstances surrounding maternal fatalities

“Yes, before we stopped actively working we used to even make monthly report on the people we have helped, the number of babies, how many are alive and how many are dead plus any complications in all the cases (TBA11, Female, 38 years, Mayuge Village)”.

#### Subtheme: Referral Process

The study explored the referral practices of Traditional Birth Attendants (TBAs), focusing on how they navigated the process of referring women to higher-level health facilities, other TBAs, or traditional healers. The findings showed that TBAs were keenly aware of the importance of referral systems, especially in situations where complications during labor or delivery exceeded their skill level or available resources. However, TBAs often faced significant challenges in executing timely referrals, primarily due to factors such as inadequate transportation, long distances to the nearest health facility, and the reluctance of families to seek formal medical care. In some cases, TBAs resorted to referrals to other TBAs or traditional healers, depending on the nature of the complication and the preferences of the family.

#### Referral to the Health Facility

The study revealed that TBAs regularly referred women to health facilities when they encountered life-threatening complications such as postpartum hemorrhage, preeclampsia, or obstructed labor. While the study found that TBAs were aware of the necessity of seeking professional medical care in emergencies, the referral process was often hindered by logistical issues, including transportation difficulties and patient reluctance. In rural areas, where TBAs typically work, accessing formal healthcare was not always straightforward due to geographical and economic barriers. TBAs reported that, in some instances, patients or their families refused to be referred to a health facility due to cultural or financial concerns. Additionally, there was a lack of formalized protocols for referral, meaning TBAs were sometimes uncertain about how to handle the referral process efficiently, especially when documentation or specific procedures were required. Despite these challenges, TBAs were committed to ensuring that women with complications received appropriate medical care whenever possible.

“No if the mother is okay I don’t go with her. The only one I go with is the one who comes in a bad condition, the one that I believe I cannot handle. I go with her because I can’t help her(TBA3, Female,53 years, Nawanvubu Village)”

“To the hospital, even if they reach at what time in the night, I discharge them, maybe if they came in at that time when I haven’t seen them and recognize them(TBA7 Male, 46 years,Buwaiswa Village)”.

#### Subtheme: TBA to TBA Referral

In cases where TBAs were unable to manage complications on their own, the study found that they would often refer women to other TBAs who they believed had more experience or expertise in handling specific obstetric conditions. This TBA-to-TBA referral system was common, particularly in areas with limited access to healthcare facilities. TBAs relied on informal networks within their community, where knowledge of other TBAs’ skills and experience was shared. While this referral process could provide a valuable safety net for women in rural areas, the study highlighted that it had its limitations. The lack of standardized practices and clinical training among TBAs meant that the quality of care at different TBA sites could vary significantly. Furthermore, some TBAs reported that when they referred patients to others in their network, they were unsure of whether the receiving TBA had the necessary expertise to address the complication, creating a sense of uncertainty in the referral chain.

“There some people who might come saying they went to the scan and where told the baby is not lying well and you check and find out its true so I keep checking her and after some time, if she came early you see it has changed Position so you can send her for a scan and confirm but if you check and it’s still not in the right position you tell her to go to one of the senior TBAs in Bugweri district (TBA13,Female, 43 years, Bukatuube Village)”

#### Subtheme: Referral to Traditional Healers

The study also found that in some cases, TBAs referred women to traditional healers, particularly for conditions believed to be rooted in cultural or spiritual causes. For example, conditions like *Nabuguma* (heat in the abdomen), which were commonly understood in the community as a result of spiritual or environmental factors, led TBAs to refer patients to traditional healers who specialized in addressing such ailments. These referrals were often made when TBAs felt that their medical knowledge or practices could not resolve the issue, or when the patient or their family preferred to seek care from a traditional healer. While this process was seen as culturally appropriate by both TBAs and the community, it also led to concerns. The study found that overlapping treatments from both TBAs and traditional healers could sometimes result in conflicting advice or practices, which in turn may have impacted patient outcomes. Moreover, the lack of integration between the formal health system and traditional healing practices sometimes made it difficult for TBAs and healthcare workers to collaborate effectively, leading to missed opportunities for holistic care.

“We refer to the traditional healers especially those with Amakiro, they calm back when they are very fine (TBA9, Female, 44, years, Mpungwe Village)”

“There are conditions which we can’t manage for example when a mother reaches 40 weeks but the baby can’t move, we refer them for spiritual intervention (TBA15, Female, 62 years, Mayuge Village)”

#### Theme: Roles and influences of social-cultural-economic factors

Social and cultural factors had significantly influenced the practices and effectiveness of TBAs. Economic hardship, such as poverty among mothers, had often forced families to rely on TBAs due to the cost of formal medical care. The influence of husbands, who might have had preferences for traditional birth practices or fear of medical institutions, had further affected the choice of care. Community support and motivation had also played a key role, as TBAs were seen as integral to local healthcare systems. These social dynamics had helped shape how TBAs operated and were perceived in their communities. The husband’s influence also played a significant role in maternal healthcare decisions. In many cases, husbands had strong preferences for where their wives would give birth, sometimes choosing traditional methods over medical interventions. Some husbands feared becoming widowers if their wives gave birth in a hospital, fearing that hospitals might be seen as dangerous or a place where death was more likely. This cultural view shaped how TBAs were perceived and how their services were used. Lastly, the motivation by community was a significant influence on TBAs. Many TBAs were driven by a deep sense of duty and commitment to their communities. They were often supported by local leaders and the general population, who valued their services.

Child Node: Poverty among mothers: Economic constraints were found to influence the decision to use TBA services. TBAs reported that many mothers could not afford the costs associated with hospital-based care, leading them to seek out TBAs for assistance with childbirth.

“ It is just that sometimes she might come in pain and tell you that she lacks transport to the facility and her husband is not around so she asks you that in case the time comes you assist her but if the husband comes back then they might go to the facility. Sometimes it is really a night and nowadays there are a lot of killings, they fear that they might be attacked on the way to hospital. Or sometimes they might come for you here to go and assist them from their home because right now I don’t have where they can give birth from (TBA6, Female, 60 years, Kigulu Village)”.

#### Child Node: Husband Influence:

Husbands played a significant role in deciding where a woman would deliver. TBAs shared that some husbands preferred the services of TBAs, often due to cultural beliefs or fear of hospital delivery, which influenced maternal care choices.

“Some mothers are brought here by their husbands to receive services and they even tell us to keep giving them feedback. Even if the situation gets worse, they still tell us to treat me (TBA5 Female, 54 years, Buwalima Village)”.

“We also deliver wives of our fellow male TBAs (name mentioned) because they can’t deliver them so they refer them to us (TBA10,Female, 32 years, Bukatuube Village)”.

#### Child node: Motivation by Community:

The community’s trust and reliance on TBAs motivated them to continue their work despite the challenges. TBAs reported that the sense of duty to the community, along with recognition of their role in maternal health, kept them engaged in providing care.

“Some people prefer us to health workers and they have supported our existence, actually when the government started fighting us, some mothers stopped going to facilities in protest and that time I had the highest number of deliveries, over 6 per day (TBA13,Female,33 years, Bukatuube Village)”

#### Child node: Customer care at TBA sites

Traditional Birth attendants offered customer care services to their clients which included a cup of tea and lunch as well as other support like washing for them after delivery.

“No may be giving her some tea. Because some people come in labor and have not had breakfast lunch or supper and sometimes I even give them some food if I have to help them regain their body strength (TBA6, Female, 60 years, Kigulu Village)”

#### Theme: Challenges faced by TBAs in providing safe and effective maternal and child Health care services

TBAs had faced numerous challenges, including official bans from health authorities and competition from both other TBAs and private clinics. They often worked under fear of sanctions, lacked support from the Ministry of Health, and struggled with resource limitations such as inadequate supplies like gloves and plastic bags. Additionally, patient negligence, such as delays in seeking care and poor antenatal care attendance, had complicated their ability to provide safe and effective services. Despite these challenges, TBAs had remained vital in areas where formal healthcare services were scarce.

#### Subtheme: Ban by the Ministry of Health.

Many TBAs operated under fear of sanctions or arrest, which stifled their ability to offer services freely. Additionally, competition among TBAs was common, both in terms of reputation and resources. Lack of support from the Ministry of Health compounded these challenges, as TBAs often lacked access to official resources and formal recognition.

“I think the government should be open and accept us to work because we are also people. They should allow us to work as we used to. If they can also get us somewhere, where we can take mother who have complications and may be improve on transportation of patients here (TBA11, Female, 38 years, Mayuge Village)”.

#### Subtheme: Threat from local district leadership

The leaders who sometimes viewed them as competitors to formal health facilities. TBA vs. TBA competition was also a concern, as rivalries between birth attendants could undermine cooperation and create confusion within the community. This competition extended to the competition with private clinics, which offered more advanced services and were often more financially supported.

“When they started talking about us on radios and telling us ‘don’t do this, don’t do that’ I decided that if someone wants my services I gave them a rule that they just call me and I go to their place and I assist them from there. Because me here I no longer have the room. I removed it. But those days I used to admit when I still had the room (TBA8, Female, 39 years, Kikuubo Village)”.

#### Subtheme: The lack of sundries by patients.

Many patients could not afford basic supplies such as gloves or plastic bags, which made it difficult for TBAs to maintain proper hygiene.

“Some patients come here with nothing, you are forced to get your own clothes like gomesi tear and give them to use for taking the baby home (TBA2, Female, 34 years, Wangobo Village)”

“There are patients come with one pair yet they are to be admitted for long which makes us end up reusing them, that I mean we wash them and keep using them (TBA1, Female, 45 years, Buguwa Village)”.

#### Subtheme: Negligence of patients.

It was reported that many patients delayed seeking care or did not follow appropriate treatment protocols. The delay to reach the TBA facility was a common issue, with women often waiting too long to seek help during labor. Poor antenatal care (ANC) visits further compounded the problem, as many women did not attend regular checkups, leading to undiagnosed complications and increased risks.

“There are these mothers who first try to deliver by themselves at home and come here when the kitani (placenta) is already out, they over bleed which makes our work hard(TBA9, Female, 44years, Mpungwe Village)”

The study found that many Traditional Birth Attendants (TBAs) expressed that they were working under a constant state of fear due to the Ministry of Health’s (MOH) restrictions on untrained birth attendants. The official stance of the MOH, which seeks to regulate maternal healthcare practices and restrict the involvement of untrained TBAs, created an environment of insecurity for many TBAs. They reported concerns about potential legal consequences and the possibility of facing penalties for providing care, despite their community’s reliance on them. Several TBAs revealed that their work was often done clandestinely, as they feared being reported or facing sanctions from health authorities. This fear of repercussions hindered their ability to deliver care confidently and often forced them to limit the scope of their services. Despite this fear, TBAs in the study also highlighted that they felt compelled to continue working because of the dire need for maternal care in rural and remote areas, where access to formal healthcare services was limited.

“When they started talking about us on radios and telling us ‘don’t do this, don’t do that’ I decided that if someone wants my services I gave them a rule that they just call me and I go to their place and I assist them from there. Because me here I no longer have the room. I removed it. But those days I used to admit when I still had the room (TBA7, Female, 46 years, Buwaiswa Village)”

Another significant finding from the study was the reported lack of support from the Ministry of Health (MOH). Many TBAs expressed feelings of abandonment and neglect by the MOH, particularly in terms of training, resources, and recognition of their role in the healthcare system. While the MOH has implemented some training programs, TBAs noted that these programs were often inconsistent, infrequent, and sometimes not accessible to everyone in need. The study revealed that TBAs frequently felt that the MOH regarded them as “informal” healthcare providers, offering limited assistance in terms of guidance, supplies, or integration into the broader healthcare system. As a result, TBAs were left to operate independently, without sufficient support, which affected the quality and safety of the care they were able to provide. TBAs in the study suggested that greater collaboration and support from the MOH could help improve the overall quality of maternal healthcare in their communities.

“I think the ministry should just support us and not fight us by the way, can they train us and take us to health facilities or even work with us. Their lack of support has made us lose morale of working S(TBA9, Female, 44, Years, Mpungwe Village)”.

“Ministry of health has not supported us instead they have tortured us yet we are the people who delivered their grandmothers etc, she cries……(TBA15, Female, 60 years, Mayuge Village)”

#### Subtheme: Threats from District Leadership

The study also highlighted that TBAs faced threats from district leadership, which compounded the fear associated with their practice. District authorities, often aligned with the MOH’s stance, sometimes issued direct threats or warnings about the illegal nature of TBA services. These threats included the risk of being fined, arrested, or facing other forms of legal action if caught providing services. Some TBAs reported that local authorities even threatened to revoke business permits or shut down their practices entirely, creating an atmosphere of constant anxiety. This tension between TBAs and district leadership stemmed from the official policy that aimed to push TBAs out of the maternal healthcare system. The study found that these threats often led to a reluctance to reach out to authorities for assistance, as TBAs feared being penalized rather than supported in their efforts to provide care.

“There is a team of people from the district which keeps coming and tells us that we are going to be arrested, we end up working while hiding (TBA6, Female, 60 years, Kigulu Village)”

“Police came and put one of our referral TBA on a track and took her to Iganga, it was a very funny gesture and I hope they won’t do it to us (TBA14, Female, 53 years, Mpungwe Village)”.

#### Subtheme: TBA vs. TBA Competition

The study revealed that competition among TBAs was another notable challenge in certain regions. In areas where multiple TBAs operated, rivalries and competition for clients were common. TBAs noted that competition sometimes led to a decrease in the quality of care, as some practitioners might feel pressured to offer faster or cheaper services to attract more clients. This rivalry could also foster a lack of collaboration among TBAs, hindering their ability to share knowledge or best practices. In some cases, competition between TBAs led to distrust within the community, where clients were divided in their preferences for one TBA over another. Despite these challenges, many TBAs stressed the importance of maintaining their relationships with their peers and found ways to cooperate, recognizing that their collective efforts were essential for improving maternal health outcomes in their areas.

“ Yare aware, we are many in Mayuge and everyone is looking for survival so some of our colleagues who have money employ midwives to keep referring mothers to them (TBA13, Female, 42 years, Bukatuube Village)”

“Some of the traditional birth attendants have agents with in the community, so find them at bore holes, community SACCO meetings encouraging mothers to go to that particular TBA, so us who don’t have, we don’t get them(TBA4, Female,38 years, Baitambogwe)”

#### Subtheme: Competition with Private Clinics

Competition with private clinics was another challenge that surfaced in the study. As private clinics offering more advanced and professional medical services began to emerge, TBAs reported losing clients who sought safer, more modern healthcare options. While TBAs were still widely trusted by their communities, many women increasingly opted for private clinics that could offer professional medical care, better facilities, and specialized staff. The study found that the rise of private clinics often left TBAs feeling marginalized and undervalued, as they were unable to compete with the resources and training available in these formal healthcare settings. However, some TBAs also mentioned that the cost of services at private clinics made them inaccessible for many women in rural or impoverished areas. Despite these challenges, the study showed that TBAs continued to serve their communities, especially in remote regions where private clinics were either scarce or non-existent.

“There are private clinics in Mayuge, they discourage mothers from coming to us but they later come after being mismanaged (TBA10, Female, 32 years, Bukatuube Village)”

#### Subtheme: Not Taking Appropriate Treatment

The study also revealed that many women did not take appropriate treatment or follow the advice given by TBAs. Some TBAs noted that patients were often non-compliant with prescribed treatments, particularly when traditional practices conflicted with the medical advice offered. This non-compliance was often due to cultural beliefs, mistrust of formal healthcare, or the influence of family members who preferred traditional healing methods over medical intervention. Additionally, the study found that some women neglected postnatal care, which was essential for monitoring both maternal and infant health after delivery. TBAs expressed frustration over this issue, as they felt that many complications could be prevented with proper follow-up care. However, they acknowledged that changing cultural attitudes and increasing education about the importance of postnatal care were critical steps in improving treatment compliance.

“You give a mother treatment to take like 4 times a day but because she delayed in the garden, she ends up taking only once, which is not right(TBA12, Female, 39 years, Nansaga Village)”

“Then there are these young mothers who come here for local herbs like to stop vomiting during pregnancy, they mix what they are given at the facility and what we give them which exacerbates more problems (TBA5, Female, 54 years, Buwalima Village)”.

Finally, the study highlighted that poor attendance at antenatal care (ANC) visits was a widespread issue. TBAs reported that many women did not attend ANC visits due to a lack of awareness about the importance of early prenatal care, financial constraints, or fear of formal medical settings. This lack of ANC visits often resulted in TBAs dealing with high-risk pregnancies that could have been better managed had the women attended regular prenatal check-ups. The study participants emphasized that the absence of proper prenatal care contributed to preventable complications during labor and delivery, such as preeclampsia and postpartum hemorrhage. TBAs expressed the need for increased community education about the importance of ANC and early intervention to ensure better outcomes for both mothers and babies.

Now there is a big challenge her, mothers come here for a full package of ANC then they also go to Mayuge HCIV, they mix the two end end up getting problems, I wonder why they cnt do one at ago(TBA8, Female, 39 years, Kikuubo Village”.

“Mothers Miss ANC visits, most of them come at the beginning and towards the last weeks, which becomes challenging to most of us ((TBA1, Female, 34years, Buguwa Village)”.

#### Theme: Contextual Factors

TBAs operate in environments with various cultural, social, and economic challenges. In some communities, traditional beliefs may discourage women from seeking modern medical care, making it difficult for TBAs to encourage hospital deliveries. Additionally, poverty limits access to essential resources, such as clean delivery kits, making safe deliveries more challenging.

#### Subtheme: Failure of Mothers to Disclose Their HIV Status

Many expectant mothers do not disclose their HIV status to TBAs due to fear of stigma or lack of awareness. This put both the TBA and the newborn at risk of transmission during delivery. Without this crucial information, TBAs were unable to take necessary precautions or provide appropriate interventions to protect both the mother and child.

“Women don’t want to tell us their HIV status especially when they come with their husbands which makes our work hard (TBA4, Female, 38 years,Waitambobwe)”

“Some women who know their status, don’t want us to tell their husbands because of fear of divorce and stigma (TBA7, Male, 46 years, Buwaiswa Village)”

TBAs often lacked access to HIV testing kits, making it difficult to identify HIV-positive mothers. Early detection is crucial in preventing mother-to-child transmission through interventions like antiretroviral therapy and safe delivery practices. Without testing kits, TBAs were unable to make informed decisions regarding the management of such cases.

“We cant keep testing every woman who comes because we can’t access enough kits, especially seasons when deliveries are many (TBA12, Female, 39 years, Nansaga Village)”

#### Subtheme: Lack of transport to health facilities for complicated Cases

In cases of complications like prolonged labour, excessive bleeding, or fetal distress, timely referral to a health facility was critical however, many TBAs operated in remote areas with poor transport infrastructure. The lack of ambulances or any means of transport put the lives of both the mother and baby at great risk.

“In case of complicated cases, I just call the boda boda man and they take to Iganga or Mayuge but I have no ambulance ……she laughs (TBA7, Male, 46 years, Buwaiswa Village)”

#### Theme: Harsh Attitude from health Workers

When TBAs refered complicated cases to health facilities, they often faced hostility from healthcare workers. Instead of collaborating with TBAs to ensure safe deliveries, some health workers dismissed or criticized them, discouraging them from making referrals in the future. This attitude created a gap between traditional and modern healthcare, ultimately affecting maternal and child health outcomes.

“You don’t know how those midwives ….. mention a name….treat us when we refer mothers there, others frit ask for money before attending to pour patients (TBA15, Female, 62 years, Mayuge Village)”

#### Theme: Lack of space for deliveries and postnatal care

Many TBAs operated from their homes or makeshift facilities without proper delivery rooms or resting spaces for mothers after childbirth. The absence of hygienic and adequately equipped spaces increased the risk of infections, postpartum complications, and poor recovery for new mothers.

“I sometimes deliver my mother’s from my neighbour’s place, just like you see, the rain disorganized my house and delivery room as well (TBA9, Female, 44 years, Mpungwe Village)”.

#### Subtheme: Unprepared Mothers for Delivery

Some pregnant women sought TBAs’ services without adequate preparation for childbirth, lacking essential supplies like clean clothes, sanitary pads, or even funds for emergency transport. This lack of preparedness increased the risk of complications and limits the ability of TBAs to provide safe and effective care.

There are some mothers who walk in here as if they have come to have lunch or even greet you, they end up having lunch with us then all of a sudden they start getting contractions, they have not come with anything and I end up giving them my gomesis.

## Discussion

Traditional Birth Attendants (TBAs) play a pivotal role in maternal and child health in many parts of the world, especially in resource-poor settings where access to formal healthcare is limited. Their contributions are most significant in rural and remote areas, where they are often the first point of contact for expectant mothers[7]. This comprehensive discussion examines the key themes and subthemes based on our findings, compares them with relevant studies, and delves into the evolving role of TBAs both in Uganda and globally[2]. The historical role and evolution of TBAs have been marked by both informal and formal learning pathways. Our findings reveal that TBAs historically learned their trade through apprenticeship, a process where novice practitioners gained hands-on experience under the guidance of more experienced birth attendants. This apprenticeship model provided TBAs with practical knowledge passed down through generations[8]. Over time, there has been an increased push for formalizing and standardizing TBA training, particularly through government-led initiatives in countries like Uganda, where the Ministry of Health (MOH) has played a pivotal role in integrating TBAs into the broader healthcare system[9]. The apprenticeship model has been the backbone of TBA practice in many African countries, including Uganda. [2]noted that the apprenticeship approach has equipped TBAs with significant practical experience in managing deliveries and complications. However, the lack of formal education means that some TBAs might not be well-prepared to handle complex situations, especially those involving complications that require advanced medical interventions. In Uganda, many TBAs have also previously worked as Village Health Teams (VHTs), which provided a foundation for basic healthcare knowledge, such as maternal health practices and hygiene[10]. VHTs are trained community health workers who bridge the gap between health facilities and rural populations. This background gives TBAs an edge in understanding community health needs and addressing local concerns, including issues related to maternal and child health however, while this previous training helps TBAs provide essential health services, the need for more standardized and comprehensive training remains critical[8].

In countries like Ethiopia and Kenya, similar trends are observed[5]. Traditional knowledge and skills, often passed down from elders to younger practitioners, are increasingly being supplemented with formal training programs to ensure better quality of care[11]. However, like Uganda, TBAs in these countries still face challenges regarding the consistency and depth of their training. Our study found that TBAs in Uganda use a combination of traditional knowledge and modern medical practices to manage obstetric conditions. Conditions such as preeclampsia, birth asphyxia, and postpartum hemorrhage are diagnosed and managed largely through physical observation and knowledge passed down from previous generations. However, TBAs often lack the tools and knowledge necessary to manage these conditions effectively, particularly when complications arise[11]. This is in line with findings by [12], who emphasized that TBAs in Uganda often perform basic interventions but are not always equipped to identify or manage high-risk pregnancies or emergency complications[13]. For example, while TBAs may recognize preeclampsia based on symptoms like swelling or hypertension, they may not have the necessary diagnostic tools or the capacity to manage severe cases, which may require referral to a healthcare facility. Similarly, in the case of postpartum hemorrhage or birth asphyxia, TBAs may administer basic interventions such as uterine massage or neonatal resuscitation, but without advanced medical equipment, the outcomes could be fatal if not addressed in a timely manner[14].The dual use of traditional and modern medicine has been well-documented across sub-Saharan Africa[15]. In Kenya and Tanzania, TBAs continue to use herbal remedies alongside modern medical treatments to manage obstetric conditions. This integration of both systems can be beneficial in settings where access to formal healthcare is limited[16]. However, this blended approach can also create confusion, especially when traditional remedies may conflict with evidence-based medical practices[17]. This is a critical issue that requires addressing through better training, supervision, and collaboration between TBAs and healthcare providers. Infection prevention and control is a cornerstone of safe maternal and child healthcare[11].Our findings suggest that TBAs in Uganda adopt various infection control practices, such as using gloves, handwashing with soap, and ensuring that waste is disposed of through burial, burning, or placement in a designated pit. These practices are essential to preventing the spread of infectious diseases, particularly in the absence of sterile conditions at birth. However, the effectiveness of these infection control practices is often undermined by resource shortages[4]. In Uganda, TBAs report difficulties in accessing basic materials such as gloves, sterilizing equipment, and antiseptics[6]. The lack of access to essential supplies hampers their ability to maintain hygienic conditions during deliveries and increases the risk of maternal and neonatal infections[7].This challenge is not unique to Uganda; other studies in sub-Saharan Africa, including Tanzania and Malawi, highlight similar issues, where TBAs struggle with the availability of sterilization equipment, leading to unsafe birthing conditions[18]. Despite these challenges, infection prevention measures such as handwashing and the use of personal protective equipment (PPE) like gloves have been shown to reduce the incidence of infection[19]. In Uganda, there has been an ongoing effort to train TBAs on proper infection control techniques, but without regular access to supplies, these measures can only go so far. Studies from other countries, such as India, also show that TBAs face significant challenges in implementing effective infection control practices due to resource constraints[2]. Social and cultural factors play a pivotal role in shaping the effectiveness of TBA services[20]. Economic hardship, cultural beliefs, and the influence of family members, particularly husbands, influence the decision to use TBA services over formal healthcare[21]. Our study found that many women choose to deliver with TBAs due to financial constraints, as the costs associated with formal healthcare can be prohibitive for families living in rural areas. These findings align with those of [3], who noted that many rural Ugandans rely on TBAs due to the high costs of transport, medical fees, and the perceived inaccessibility of health facilities[3].

In Uganda, husbands often exert significant influence over the choice of delivery method, with many preferring the services of a TBA over a hospital-based delivery due to fear of medical interventions, perceived cultural appropriateness, or mistrust of the formal healthcare system[22]. This is consistent with findings from other African countries, including Nigeria and Ethiopia, where the influence of male partners plays a major role in maternal health decision-making[23]. Community support is also a significant factor in the effectiveness of TBAs, as they are viewed as trusted figures within their localities[23]. In rural Uganda, TBAs are often seen as integral to the community’s well-being, and their services are deeply rooted in local traditions and customs[22]. TBAs face numerous challenges in providing safe and effective care. Our findings reveal that many TBAs work under fear of legal consequences, especially with increasing governmental bans on their practice[2]. These bans, often imposed by the Ministry of Health (MOH), are meant to protect mothers and children from unsafe deliveries[20]. However, these bans have left TBAs working in a legal grey area, without formal support from the health sector[9]. Competition from other TBAs, private clinics, and health facilities has further complicated the situation[12]. While some TBAs view other practitioners as rivals, others have formed networks to provide mutual support and share resources. The lack of governmental support is another pressing issue, with TBAs reporting minimal interaction with health authorities, leaving them without training updates, resources, or recognition for their contributions[12]. Moreover, inadequate supplies, including gloves, disinfectants, and sterile materials, make it difficult for TBAs to provide optimal care. This issue is particularly acute in Uganda, where rural areas are often underserved by formal healthcare infrastructure[18].

While TBAs in Uganda and other countries face these challenges, their continued relevance cannot be understated. As our study highlights, TBAs remain essential healthcare providers in rural and remote areas. However, addressing the challenges they face, particularly concerning training, resources, and integration into the formal healthcare system, is crucial to improving maternal and child health outcomes.

In conclusion, TBAs in Uganda and other countries continue to play a vital role in maternal and child health, especially in rural and underserved regions. However, their practices are influenced by a variety of factors, including historical training models, the integration of modern medical practices, infection control measures, and socio-cultural dynamics. The challenges they face, particularly related to resource shortages, competition, and lack of formal support, must be addressed through better training, resource allocation, and stronger partnerships with formal health systems.

Global studies on TBAs, including those in Ethiopia, Kenya, and India, show that while TBAs can provide essential services, they require more formal support, training, and integration into the broader healthcare framework to reduce maternal and neonatal morbidity and mortality. Addressing these issues is crucial for optimising the role of TBAs and improving the overall health outcomes of the populations they serve.

## Data Availability

The transcripts used and/or analyzed during the current study are available from the corresponding author upon reasonable request.
